# An integrated process for the extraction of fuel and chemicals from marine macroalgal biomass

**DOI:** 10.1038/srep30728

**Published:** 2016-07-29

**Authors:** Nitin Trivedi, Ravi S. Baghel, John Bothwell, Vishal Gupta, C. R. K. Reddy, Arvind M. Lali, Bhavanath Jha

**Affiliations:** 1 Division of Marine Biotechnology and Ecology, CSIR- Central Salt and Marine Chemicals Research Institute, Bhavnagar, Gujarat, 364002, India; 2 Academy of Scientific & Innovative Research (AcSIR), New Delhi, India; 3 School of Biological and Biomedical Sciences, Durham University, South Road, Durham, DH1 3LE, UK; 4 DBT-ICT Centre for Energy Biosciences, Institute of Chemical Technology, Mumbai, 400019, India

## Abstract

We describe an integrated process that can be applied to biomass of the green seaweed, *Ulva fasciata*, to allow the sequential recovery of four economically important fractions; mineral rich liquid extract (MRLE), lipid, ulvan, and cellulose. The main benefits of our process are: a) its simplicity and b) the consistent yields obtained from the residual biomass after each successive extraction step. For example, dry *Ulva* biomass yields ~26% of its starting mass as MRLE, ~3% as lipid, ~25% as ulvan, and ~11% as cellulose, with the enzymatic hydrolysis and fermentation of the final cellulose fraction under optimized conditions producing ethanol at a competitive 0.45 g/g reducing sugar. These yields are comparable to those obtained by direct processing of the individual components from primary biomass. We propose that this integration of ethanol production and chemical feedstock recovery from macroalgal biomass could substantially enhance the sustainability of marine biomass use.

Falling fossil fuel reserves and growing atmospheric CO_2_ emissions have made the development of renewable biofuels a global priority^1^. One possible source of renewable biomass is the macroalgae, or seaweeds, which are increasingly recognised as an attractive and competitive commercial biofuel crop^2^,^3^,^4^,^5^. Seaweeds grow fast, do not compete for land with food crops and – as a traditional source of food, feed, fertilisers and phycocolloids - are already farmed on large scales in a number of Asian countries.

The worldwide seaweed production has been estimated at about 24 million tonnes fresh weight per annum^6^, with a market value of over USD 7.4 billion. A little over USD 1 billion of this comes from the algal colloid industry, in which agars and carrageenans are extracted from red seaweeds and alginates are extracted from brown seaweeds. Phycocolloids are modified polysaccharides that are suitable for fermentation into bioethanol, and many studies have already assessed the production of either ethanol^4^,^7^ or butanol^8^ from phycocolloid species. In addition, red seaweeds have recently been evaluated for their biorefinery potential in order to integrate the production of bioethanol with high value products from red algal biomass^9^,^10^.

These advances notwithstanding, the phycocolloid industry may not be the only place to look for biofuel biomass. Phycocolloid farming has almost doubled its market value over the past decade^11^,^12^ and supports a number of rural economies. Consolidation of this economic development, together with issues of biosecurity and resource sustainability, make it desirable, therefore, to diversify and explore new seaweed feedstocks.

The floating green seaweeds are emerging as strong candidates in this search: green seaweeds have extremely high productivity and are widely distributed across diverse geo-climatic conditions^13^,^14^,^15^. Indeed, their growth is so rapid and so widespread that they have contributed to harmful green tides in places as far apart as China, Europe and the US, posing severe environmental and economic threats to both aquaculture and tourism^16^. According to reliable estimates, for example, the clean-up costs for green tide in the Yellow Sea (triggered by the release of effluents from aquaculture farms of the red seaweed, *Porphyra*) ran to around USD 30 million, which may be compared to a total profit of only USD 53 million from *Porphyra* cultivation in the region in the first place^17^.

There are, therefore, pressing reasons to consider how green seaweeds could supply biofuels and other products in economically useful amounts. Accordingly, we now describe a simple, sequential process that can extract multiple components from the green species *Ulva fasciata*. We focus on four particular components with known applications: first, the MRLE that contains nutrients that are suitable for human and animal food supplements^18^. Second, the small (~1–3% of dry wt), but nutritionally important, lipid fraction that is rich in essential polyunsaturated fatty acids^18^,^19^,^20^. Third, the major colloid fraction, which in *Ulva* spp. comes primarily in the form of ulvan, a water soluble sulphated polysaccharide with potential applications in the food, pharmaceutical, agricultural, and chemical industries^14^,^21^,^22^. Fourth, and finally, the cellulose fraction, which can be used as a feedstock for bioethanol production. Overall, our process demonstrates one possible way in which the biofuel production from seaweeds might be made more efficient. This has obvious and immediate implications for the potential costs and labour involved in sustainable marine resource use.

## Results and Discussion

### Multiple fractions can be extracted in a sequential and scalable fashion from *U. fasciata*

**Figure 1:**
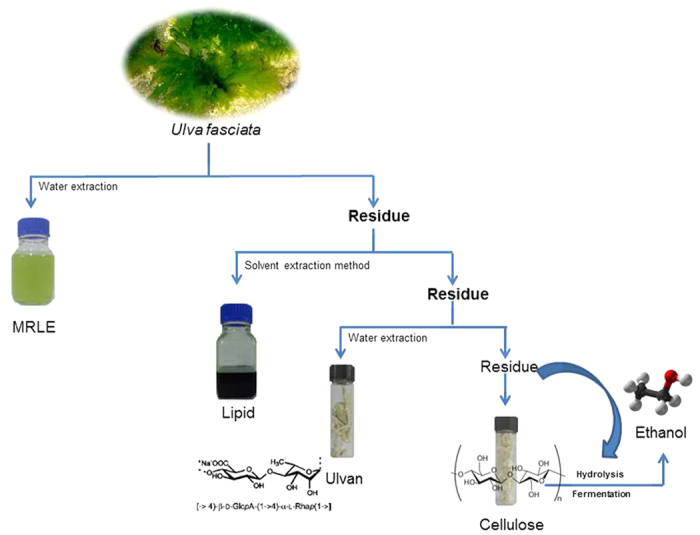
A schematic representation of process integrating bioethanol production with sequential extraction of products from fresh *U. fasciata* feedstock (Photographs taken by Mr. NitinTrivedi, one of the coauthors of this article).

Four fractions (MRLE, lipid, ulvan and cellulose) were extracted from fresh biomass of *U. fasciata* using our integrated sequential process (Fig. 1; cf. Methods and materials). The optimal order in which to extract these four fractions was arrived at after a series of preliminary trials, and two questions were then asked: 1) Were the sequential yields at a range of scales comparable to the yields from direct individual extractions? and 2) What were the efficiency gains from our sequential process?

**Figure 2:**
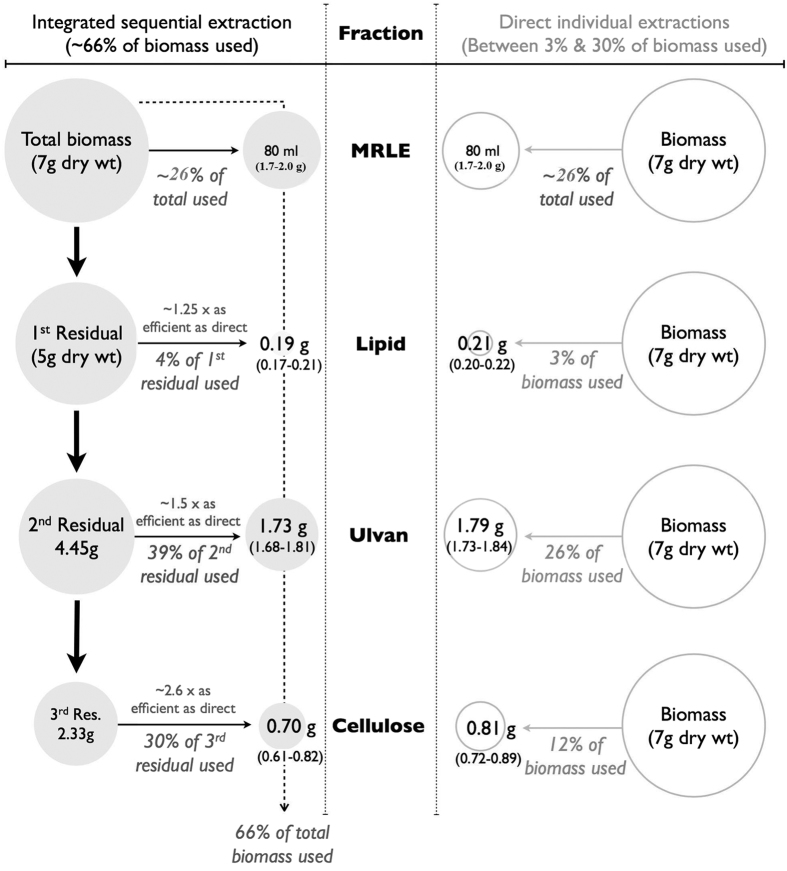
Sequential extraction yields are comparable to those from direct extractions. Yields are given as mass extracted from 50 g FW *U. fasciata* (equivalent to 7 g DW, as *U. fasciata* is 86% water). Efficiency gains are indicated.

**Figure 3:**
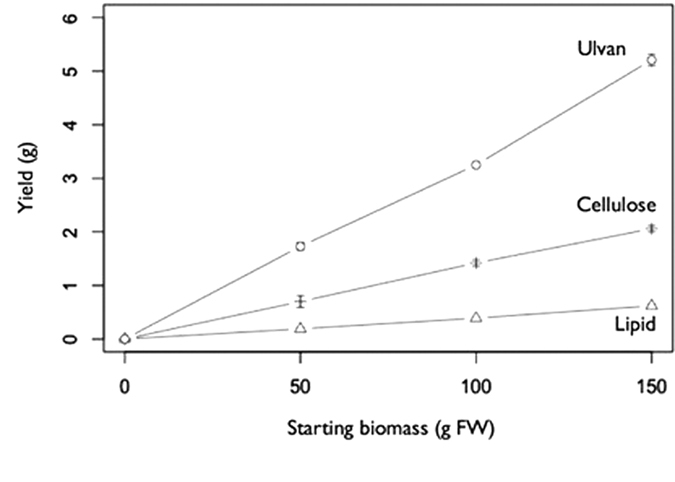
Sequential extraction yields scale with starting biomass in a linear fashion. Values shown are for three replicates per condition.

**Figure 4:**
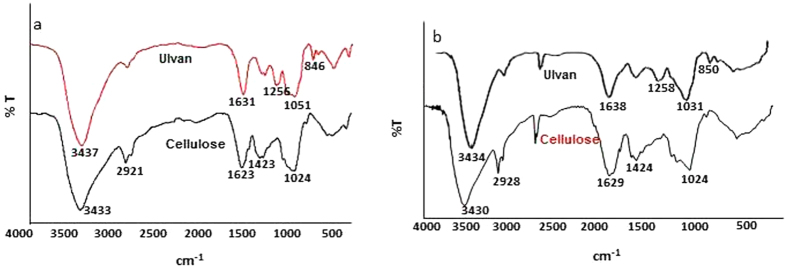
FTIR spectra of ulvan and cellulose extracted in (a) integrated and (b) direct process from *U. fasciata*.

Our first question can be answered quantitatively: we found that the yields of lipid, ulvan and cellulose from our sequential process were not significantly different to their yields from direct individual extractions (Fig. 2), we observed that yields of lipid, ulvan and cellulose increased in a linear fashion with starting biomass (Fig. 3), and we noted that the purity of fractions obtained from our sequential process were indistinguishable from the purity of fractions obtained from direct extraction (Fig. 4, cf. below for FTIR data and Table 1 for FA profiling).

Our second question must be answered qualitatively, as efficiency gains are dependent on process costs, which change year-on-year. These efficiency gains come from two main aspects of our process. First, our sequential process concentrates the extractable fractions into the residual biomass left after each step (Fig. 2). This means that our sequential extractions become increasingly efficient when compared to individual direct extractions. For example, in our comparison the direct extraction of cellulose starts from 7 g of dry weight (DW) biomass (Fig. 2); in our sequential extraction, however, we start from around 2–3 g of biomass in which cellulose has already been concentrated by the prior removal of MRLE, lipid, and ulvan. This reduces the chemical consumption for cellulose extraction to around 30–40% of that needed for direct extraction, reducing both the economic and environmental costs of biomass processing.

Second, our sequential process delivers four fractions, rather than one. All four of these fractions are economically useful and confer additional efficiency gains, as follows.

### Fraction 1: Mineral rich liquid extract reduces the cost of dewatering seaweed biomass

**Figure 5:**
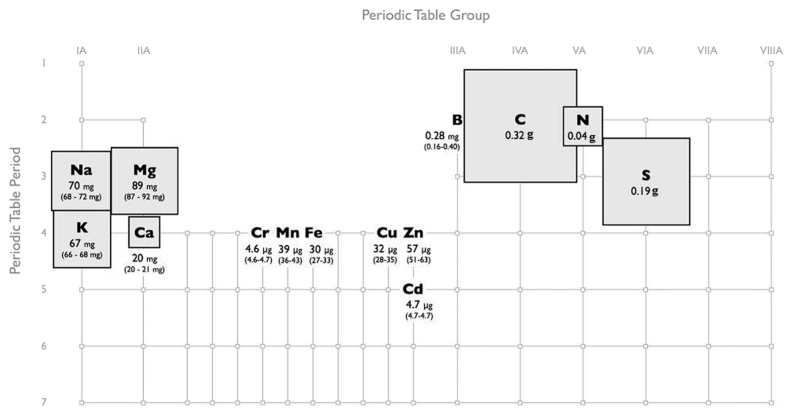
Elemental content of MRLE fraction. The elements recovered are shown in their representative locations on the Periodic Table and the size of each square is proportional to the amount recovered. Yields given are amounts found in 80 mL of MRLE (our sequential process takes 50 g FW *U. fasciata* and adds 100 mL of water to produce 80 mL of MRLE). Note that C, N and S are given as g of MRLE on DW, and not g of starting biomass.

Seaweed biomass is ~90% water. The costs of dewatering are considerable, but could be reduced if intra- and extra-cellular minerals could be removed along with the water. Our sequential process does this, and the mineral, carbon, nitrogen, and sulphur composition of the resulting MRLE is summarized in Fig. 5. Similar extracts show promise as liquid fertilisers: mungo bean plants (*Vigna mungo* L) treated with a foliar spray derived from *Ulva reticulata* (Forsk.) showed improved biochemical profiles (chlorophyll a & b, protein, sugar and starch)^23^ and *U. lactuca* extracts show similar effects on the growth of tomato seedlings^24^.

### Fraction 2: Lipid extraction gives fatty acids with desirable PUFA ratios

Our sequential process allows lipids to be extracted from biomass after MRLE removal, with ~2.7% of the total DW being recovered as lipid (0.17 to 0.21 g from a starting sample of 7 g DW; Fig. 2). This is consistent with earlier reports^25^ that describe lipid yields of between 1.83 and 2.03% in *U. fasciata, U. reticulata* and *U. rigida*. The fatty acid profiles of our *U. fasciata* lipid fractions match profiles we have previously obtained by direct extraction and are summarized in Table 1.

Although the green seaweeds have relatively low lipid contents, this is balanced by the fact that their fatty acid compositions are high in C18 (linoleic and alpha-linolenic)^20^ and low in C20 PUFAs; a combination that has been associated with the prevention of cardiovascular diseases, osteoarthritis and diabetes^26^. Overall, unsaturated FAs (mono- and poly-unsaturated) accounted for ~60% of the total fatty acids, and include several important long chain PUFAs, such as eicosapentaenoic acid (EPA, C20:5 n-3) and arachidonic acid (AA, C20:4 n-6).

### Fraction 3: High ulvan yields provide a unique biomass advantage

In the third stage of our sequential process, residual biomass (now down to 4.45 g DW from a 7 g start; Fig. 2) was processed for ulvan extraction. Ulvan comprised ~39% of the residual, or ~25% of the starting DW biomass, which is consistent with previous estimates of ulvan between 8 and 29% of DW across a range of green seaweeds^14^,^22^.

Ulvan is a sulphated polysaccharide that has demonstrated a range of desirable biological properties, having been shown to act as an anticoagulant, an antioxidant, an anti-inflammatory agent, an antiviral and an anti-proliferative agent^27^,^28^. It has also been used as an ingredient in functional foods and novel drugs^14^,^29^, as well as in a number of biomedical applications, both as an antihyperlipidemic agent and as a source of scaffold materials^30^,^31^.

Because of its importance, we verified the identity and purity of ulvan in our extract by confirming the presence of the characteristic FTIR peaks for the sulfate group on the C3 of ulvan’s rhamnose residues (L-Rha 3S)^32^,^33^: S=O stretching at 1256 cm^−1^ and C-O-S stretching at 846 cm^−1^ (Fig. 4a; cf. purity against the spectrum from directly extracted ulvan in Fig. 4b).

### Fraction 4: A cellulose-rich residual may be hydrolysed enzymatically to give bioethanol

The residual biomass left after ulvan extraction came to ~2.33 g DW. Enzymatic hydrolysis of this residual biomass, followed by fermentation of the hydrolysate by *Saccharomyces cerevisiae,* gave an ethanol yield of 440 mg/g reducing sugar. Perfectly efficient fermentation would generate 514 mg of ethanol for every 1 g of reducing sugar, so this represents a conversion efficiency of ~86%. It also bypasses any need for a separate cellulose extraction step, thereby reducing chemical inputs and making the overall process more economically and environmentally viable. And, finally, direct fermentation utilises the nitrogen and carbon that is already present in the residual biomass, so that the fermentation broth does not need the usual supplementation with these nutrients.

Alternatively, a final cellulose extraction step may be carried out if desired; we note that the chemical consumption for this final cellulose extraction will be only ~30–40% of that usually seen in direct cellulose extractions, because of the concentration of cellulose into a smaller residual biomass (i.e. extracting from ~2.33 g rather than from 7 g DW; cf. Fig. 2). This final extraction yielded 0.61 to 0.82 g of cellulose from our residual biomass samples (identity and purity confirmed by FTIR^4^; cf. Fig. 4). This is equivalent to ~30% of the residual biomass or ~10% of the starting 7 g DW biomass (Fig. 2), and is comparable to cellulose yields of 0.72 to 0.89 g from direct extraction. This final cellulose extraction will, of course, improve the efficiency of cellulose fermentation. Enzymatic hydrolysis of our extracted cellulose gave a reducing (monosaccharide) sugar yield of 930–942 mg/g cellulose, and fermentation of this hydrolysate gave an ethanol yield of 450 mg/g reducing sugar. Both the reducing sugar yield and our fermentation efficiency compare favourably to previous studies: our reducing sugar yield of ~940 mg/g cellulose is higher than the 870 mg/g cellulose reported for *Gracilaria verrucossa* and the 546 mg/g cellulose for *Gelidiella acerosa*^34^,^35^. Similarly, our ethanol yields compared favourably with those reported for brown^36^,^37^ and red seaweeds^38^,^39^, with our 450 mg ethanol/g reducing sugar being comparable to the high value of 470 mg ethanol/g reducing sugar obtained from the red seaweed, *Gracilaria*^40^.

### Advantages of the integrated process over direct extraction process

Biorefinery approaches to improve algal biomass use have a relatively recent history^41^,^42^,^43^. The biorefinery process that we demonstrate in this study extracts economically important chemicals (MRLE, lipid, ulvan and cellulose) without compromising individual yields and uses fewer reagents than if each component were extracted individually. The economic gains obtained from this improvement in efficiency could therefore potentially reduce the costs incurred in bioethanol production.

### Estimated product yields on processing one tonne fresh *U. fasciata*

It is evident from the computation of data that one tonne of fresh *U. fasciata* biomass could give approximately 37 kg MRLE, 3.8 kg lipid, 34.6 kg ulvan and 14.0 kg cellulose on DW basis. Further, 14.0 kg cellulose on hydrolysis and fermentation produce 5.85 kg ethanol. The quantification of products were carried out from the DW given in Fig. 2.

## Conclusions

The present study describes a simple sequential processes for the production of biofuel (bioethanol) and various commodity chemicals from the biomass of the common green seaweed *U. fasciata*. The distinct advantage of our process is that the sequential concentration of residual biomass by successive extraction steps reduces reagent demand in the downstream extraction and processing of fractions. Fraction recovery compares well with direct individual extraction processes, so our findings represent a significant improvement in biomass processing technology. Our process can inform the design and construction of viable biorefineries running on marine biomass, which – *inter alia* - can mitigate nuisance algal blooms, improve resource sustainability, and slow the spread of invasive biomass.

## Materials and Methods

### Materials

All analytical chemicals, media components and reagents used in this work were purchased from Sigma Aldrich (U.S.A.) and Himedia laboratories (Mumbai, India).

### Collection of algal sample

*U. fasciata* Delile was collected from the intertidal belt along the Adri coast of Gujarat, India (20°57′N, 70°16′ E) in October 2013. The alga was transported to the laboratory in wet tissue towels in an ice box and thoroughly brushed and cleaned in autoclaved seawater to remove salts, epiphytes and debris before being used for sequential or individual extractions. All extractions were conducted in replicates of three and results are presented as means and ranges.

### Mineral rich liquid extract

Fresh algal sample was mixed with distilled water (100 mL water per 50 g fresh weight) and ground in a mixer to obtain a fine paste-like suspension. The semi-solid paste was then centrifuged for 10 min at 8000 rpm in a benchtop centrifuge (Eppendorf 5804R, Germany). The clear supernatant was collected and the residual biomass passed to downstream processing (Fig. 2). Mineral analysis of the liquid extract was carried out using inductively coupled plasma atomic emission spectroscopy (Perkin-Elmer, Optima 2000, USA). In order to determine the CNS content, the clear supernatant was dried to constant weight at 50 °C. Total elemental (CNS) content was then determined using a CHNS Elemental Analyzer (Perkin-Elmer Model 2400, USA), calibrated with acetanilide as a reference standard.

### Extraction of total lipid

The residue that remained after MRLE extraction was processed to extract total lipids following the method of Bligh and Dyer (1959)^44^. For this, the residue was added to solvent mixture (1:2 chloroform: methanol; 100 mL per ~5 g dry residual biomass) and homogenized by vortexing. The sample was centrifuged at 4000 rpm at 4 ^o^C for 20 min in a benchtop centrifuge and the supernatant was removed and stored; this process was repeated 2–3 times until the supernatant was clear. The supernatants from each sample were then pooled and filtered through 44 μm Whatman filter paper. The filtrate (= lipid solution) was then water washed followed by centrifugation at 4000 rpm for 5 min at 4 ^o^C in a benchtop centrifuge. After centrifugation, the lower layer of lipid was collected and dried in a rotary evaporator to determine the weight.

To assess purity, fatty acids were profiled according to the method of Kumari *et al*.^20^. Briefly, lipid samples were mixed with 1 mL of 1% NaOH in methanol and heated for 15 min at 55 °C. 2 mL of 5% methanolic HCl were then added, and the resulting mixture was heated again for 15 min at 55 °C, before the final addition of 1 mL of milli-Q water^45^. FAMEs were extracted in hexane and Nonadecanoic acid was used as an internal standard. The GC–MS analysis of FAME samples was carried out on a QP-2010 gas chromatography–mass spectrometer (GC-2010 coupled with GC–MS QP-2010) equipped with an auto sampler (AOC-5000) from Shimadzu (Japan) using a RTX-5 fused silica capillary column, 30 m 0.25 mm 0.25l (Rastek).

### Extraction of Ulvan

Following lipid extraction, the residual biomass was further processed to extract ulvan using the method described by Jaulneau *et al* 32. Residual biomass was mixed with distilled water (100 mL per ~4.45 g dry residual biomass), autoclaved for 2 h at 90 °C, and the resulting solution was mixed and filtered through muslin cloth. The filtrate was precipitated with 2–2.5 volumes of chilled isopropyl alcohol for 24 h at −40 °C. The pellet formed after precipitation was filtered and dried and the dried sample was characterized using Fourier transform infrared (FT-IR) spectroscopy (Perkin-Elmer spectrum GX FT-IR system; Perkin-Elmer, USA) between 4000–400 cm^−1^.

### Cellulose extraction and characterization

After ulvan extraction, the leftover biomass was used for cellulose extraction using the method described by Mihranyan *et al*.^46^. Residual biomass was soaked in acetate buffer (100 mL per ~2.5 g dry residual biomass) containing 1.17 g NaClO_2_ for bleaching at 60 °C for 6–8 h. The bleached algal residues were neutralized by washing with water, and residues were then soaked in 50 mL of 0.5 M NaOH solution at 60 °C for 8–10 h. The alkali-treated biomass was then washed with water until its pH became neutral (~7.0). After washing, the biomass obtained was resuspended in 50 mL hydrochloric acid (5% v/v) and heated to 100 °C until boiling started. The resulting slurry was kept overnight at room temperature before being washed with water, filtered and dried to give the final cellulose fraction (characterized by FTIR as described earlier).

In order to realize the quality and quantity of all four products, the experiment has also been carried out with 100 g and 150 g of fresh biomass to verify the feasibility of the process under similar experimental conditions.

### Enzymatic hydrolysis and bioethanol production

Enzymatic hydrolysis of either extracted cellulose or the residual biomass obtained after ulvan extraction, and their subsequent fermentation, were carried out under optimized conditions as described in our previous work^4^. Saccharification was carried out with commercial cellulase 22086 (Novozyme, Denmark) (2% v/v concentration) at 45 °C for 36 h. For extracted cellulose (but not post-ulvan extraction residue), the hydrolysate was then enriched with peptone (5 g/L) and yeast extract (3 g/L). For both starting materials, hydrolysate was then fermented with fresh *Saccharomyces cerevisiae* (MTCC No. 180) culture (10^9^ CFU/mL) for 12 h at 28 ± 2 °C and a shaking speed of 120 rpm. For ethanol analysis, 5 mL of fermentation broth was withdrawn and centrifuged at 10,000 rpm at 4 °C in a benchtop centrifuge. The resulting supernatant was analyzed using a GC-MS (Shimadzu QP 2010) coupled to a head space (AOC-5000) analyzer^47^. Ethanol production in the fermentation broth was confirmed by retention time and mass fragmentation, and HPLC grade ethanol was included as a reference standard.

## Additional Information

**How to cite this article**: Trivedi, N. *et al*. An integrated process for the extraction of fuel and chemicals from marine macroalgal biomass. *Sci. Rep.* **6**, 30728; doi: 10.1038/srep30728 (2016).

## Figures and Tables

**Table 1:** Fatty acid composition of *Ulva fasciata* (% of total fatty acid methyl esters; FAMEs).

FAs	*U. fasciata*
**C14:0**	0.55–0.57
**C16:0**	37.4–39.8
**C18:0**	0.82–0.93
**C20:0**	0.16–0.18
**C22:0**	1.0–1.1
**C16:1(n-7)**	0.98–1.05
**C18:1(n-9)**	21.2–21.7
**C18:2(n-6)**	7.5–7.8
**C18:3(n-6)**	0.80–0.91
**C20:3(n6)**	0.47–0.49
**C20:4(n-6)**	0.47–0.55
**C20:5(n-3)**	0.32–0.52
**C18:3(n-3)**	24.8–28.3
**SFAs**	40.0–42.5
**MUFAs**	22.2–22.7
**PUFAs**	35.0–37.8
**n6FAs**	9.2–9.7
**n3FAs**	25.3–28.6
**n6/n3**	0.32–0.38
**UI**^**a**^	121.4–129.2

UI^a^ (unsaturation index) was calculated by multiplying the percentage of each fatty acid by the number of double bonds followed by summing up these contributions.
